# α-Olefin Oligomerization Mediated by Group 4 Metallocene Catalysts: An Extreme Manifestation of the Multisite Nature of Methylaluminoxane

**DOI:** 10.3390/polym17010046

**Published:** 2024-12-28

**Authors:** Francesco Zaccaria, Antonio Vittoria, Giuseppe Antinucci, Roberta Cipullo, Vincenzo Busico

**Affiliations:** Department of Chemical Sciences, Federico II University of Naples, via Cinthia, 80126 Napoli, Italy; francesco.zaccaria@unina.it (F.Z.); antonio.vittoria@unina.it (A.V.); busico@unina.it (V.B.)

**Keywords:** oligomerization, metallocenes, poly(alpha-olefins), methylaluminoxane, molecular weight distribution, Schulz–Flory

## Abstract

Group 4 metallocenes are competent catalysts for the oligomerization of higher α-olefins. Among the many chemical and physical variables of importance in the process, one is the choice of cocatalyst (activator). The impact of various activators on the performance of a representative catalyst, (nBuCp)_2_ZrCl_2_, in the oligomerization of 1-octene was thoroughly investigated; in particular, the molecular weight distribution (MWD) of the oligomers was determined by means of high-resolution high performance liquid chromatography (HR-HPLC). Unexpectedly, a bimodal MWD was highlighted when the precatalyst was activated with methylaluminoxane (MAO), whereas a single Schulz–Flory (SF) MWD was observed with borate salts. The presence of Al centers with different Lewis acidity in the complex and ill-defined structure of MAO is well known, and the broadening effects on the MWD of olefin polymerization products made with metallocene/MAO catalyst systems have been reported before. However, to the best of our knowledge, clear HR-HPLC evidence of two active species resulting from activation with MAO of one single zirconocene precursor, yielding two discrete SF product distributions, is unprecedented. By varying the polarity of the reaction medium, we managed to modulate the MWD of the oligomers from bimodal to monomodal, even with MAO, thus demonstrating that ion pairing effects are behind these unusual findings.

## 1. Introduction

Catalytic oligomerization of higher α-olefins has gained great industrial and scientific importance as a way to convert simple unsaturated hydrocarbons into high-value products [1]. Out of the approximately 5 million metric tons produced annually worldwide, the lighter fractions (e.g., 1-butene, 1-hexene) are utilized mainly as comonomers for the production of linear low-density poly(ethylene) [2,3], whereas the heavier fractions (e.g., 1-octene, 1-decene, or mixtures thereof) are oligomerized to short-chain poly(α-olefin)s (PAO) [4,5].

PAO are non-toxic, non-polar products with low volatility that find relevant applications, as such or as intermediates, in several fields as detergents, surfactants, plasticizers, drag reducing agents, and especially lubricants, to name a few. Compared with silicon-based alternatives, PAO lubricants feature superior performance (in terms of viscosity index, evaporative loss, pour point, thermal oxidative stability, and more) and a lower environmental impact [6,7]. Industrial production is multistep, beginning with catalytic oligomerization, followed by the hydrogenation of unsaturated products, then the distillation of separate more volatile fractions from heavier fractions, and finally functional additivation [4,5,6,7]. A wide range of oligomerization catalysts and reaction conditions can be employed in the first step; the two main classes are (i) Lewis acids, such as boron trifluoride or aluminum trichloride, and (ii) molecular organometallic catalysts (e.g., Group 4 metallocenes or ‘post-metallocenes’) [1,4,5,8]. The latter are generally preferred in view of their superior activity, selectivity, and environmental friendliness.

Catalyst system activity and molecular weight capability greatly depend on the (pre)catalyst structure [1,5,9,10,11,12], choice of cocatalyst (activator) [13,14,15,16,17,18,19], and reaction conditions [13,14,15,16,17,18,19,20,21]. In particular, it has been reported that different activators, like, e.g., methylaluminoxane (MAO) [15,16], MAO/tri-*iso*butylaluminum (TIBA) [19], or borate salt/TIBA combinations [17], as well as precatalyst/activator mole ratios, can affect the average molecular weight (MW) and molecular weight distribution (MWD) of the produced oligomers, even though the mechanistic origins of these observations have not been fully identified. Precise control over said parameters is key for designing materials with enhanced or specialized performance for various applications, including lubricants, adhesives, and specialty chemicals [4,5].

Herein we studied the oligomerization of bulk 1-octene at 100 °C mediated by a commercial oligomerization catalyst, namely (*n*BuCp)_2_ZrCl_2_ (**1**) [22,23,24,25], in combination with a variety of activators including MAO [26]; ‘modified’ MAO (MMAO-12) [27,28]; the reaction product of MAO with 2,6-di-*tert*butylphenol (MAO/BHT) [29,30,31]; mixtures of TIBA with *N,N*-dimethylanilinium borate, [HMe_2_N(C_6_H_5_)][B(C_6_F_5_)_4_] (TIBA/AB) [32,33]; or trityl borate, [(C_6_H_5_)_3_C][B(C_6_F_5_)_4_] (TIBA/TTB) [32,33]; as well as a recently developed Al-alkyl borate salt of composition {[(*iso*butyl)_2_(Me_2_N(C_6_H_5_))Al]_2_(*μ*-H)}[B(C_6_F_5_)_4_] (AlHAl) [34,35,36,37]. Unexpectedly, product characterization by means of high-resolution high performance liquid chromatography (HR-HPLC) revealed MWD consists of two Schulz–Flory (SF) functions in the case of the MAO-based activators, whereas a classical single-center behavior was observed with borate activators. These intriguing findings and their origin are presented and discussed in the following sections.

## 2. Materials and Methods

### 2.1. Materials

All manipulations of air-sensitive compounds were conducted under argon or nitrogen using Schlenk techniques and/or MBraun LabMaster 130 glove boxes. Morevoer, (*n*BuCp)_2_ZrCl_2_ (Sigma-Aldrich, MO, USA), MAO (Lanxess, Cologne, Germany), TIBAL (Lanxess, Cologne, Germany), TMA (Lanxess, Cologne, Germany), MMAO-12 (Merck, Darmstadt, Germany), BHT (Merck, Darmstadt, Germany), TTB (Acros, Milan, Italy), and AB (Merck, Darmstadt, Germany) were purchased and used as received. Toluene (Romil, Cambridge, UK), 1,2-difluorobenzene (Romil), and 1-octene (Merck) were purchased and purified by passing through a mixed-bed-activated Cu/4-Å mol-sieve column in an MBraun SPS-5 unit (final concentration of O_2_ and H_2_O < 1 ppm_v_). AlHAl [34] and MAO/BHT [29] were synthesized according to the procedures detailed in the literature.

### 2.2. 1-Octene Oligomerization

Experiment with 1-Octene oligomerization were carried out in a Symyx (now Unchained Labs) Parallel Pressure Reactor (PPR48) High Throughput Experimentation platform, adapting the established protocols as follows [38,39,40,41,42]. Prior to the execution of a reaction library, the PPR modules underwent ‘bake-and-purge’ cycles overnight (8 h at 85 °C with intermittent dry N_2_ flow) to remove any contaminants and leftovers from previous experiments. After cooling to glove-box temperature, the stir tops were taken off, the 48 cells were fitted with disposable 10 mL glass inserts (pre-weighed in a Mettler-Toledo (OH, USA) Bohdan Balance Automator) and titanium stir paddles, after which the stir tops were put back in place. Next, the modules were thermostated at 45 °C, and the proper volumes (0.1 mL) of toluene scavenger (MAO for MAO-based activators; TIBA for borate-based activators) and monomer (1-octene, 4.1 mL) were loaded into each reaction cell. The modules were then pressurized with nitrogen (50 psi) to seal the rubber septa of the reaction cells and heated to reaction temperature (100 °C) with stirring at 400 rpm. Once thermal equilibrium was reached, the proper volumes of the toluene solutions of the precatalyst (typically, 50 nmol in 0.8 mL) and activator (25 mM for MAO-based activators; 1–2 mM for borate-based activators) were sampled out and injected into each cell. After the desired time (5–60 min), the reactions were quenched with an overpressure of dry air. Once all the cells were quenched, the modules were cooled down and vented, the stir-tops were removed, and the glass inserts containing the reaction phase were taken out and transferred to a Genevac (Ipswich, UK) EZ2-Plus centrifugal evaporator, where all volatiles were distilled out and the oligomers were thoroughly dried overnight (75 °C, 5 mbar). Monomer conversion was measured by robotically weighing the dry oligomers while still in the reaction vials, subtracting the pre-recorded tare.

### 2.3. HR-HPLC Characterization

HR-HPLC traces of all 1-octene oligomerization products were recorded using an Agilent (Santa Clara, CA, USA) 1260 Infinity II setup, equipped with a refractive index (RI) detector and two Agilent InfinityLab Oligopore 6 μm columns with a linear MW operating range from 0.1 to 3.3 kDa.

The samples (~3 mg) were dissolved at RT in ~3 mL of THF containing BHT (0.4 mg/mL) as a stabilizer. After dissolution, the samples were sequentially injected into the column line at 35 °C and a flow rate of 1.0 mL min^−1^. Calibration was carried out with the universal method using two different sets of 10 monodisperse polystyrene samples.

Detailed analysis of the weight fractions of the oligomers was carried out using the “Peak Analyzer” routine of the Origin^®^ software suite (version 9.1), by integrating all peaks of the HR-HPLC traces. Best-fit simulations of the product distributions were carried out, assuming one or two SF functions to obtain the lowest $\chi_{R}^{2}$ value.

### 2.4. ^1^H NMR Characterization

The 400 MHz ^1^H and ^13^C NMR spectra of the selected oligomer samples were recorded with a Bruker (MA, USA) Advance III 400 spectrometer equipped with a 5 mm high-temperature cryoprobe and a robotic sample changer with pre-heated carousel (24 positions), following protocols reported elsewhere [43,44]. The samples (~25 mg) were dissolved at 120 °C in tetrachloroethane-1,2-*d*_2_ (0.7 mL) containing BHT as a stabilizer and loaded in the carousel maintained at the same temperature. The spectra were taken sequentially with automated tuning, matching, and shimming. The operating conditions were as follows: 90° pulse; acquisition time, 2.0 s; relaxation delay, 10.0 s; 16 transients.

Resonance assignment was based on the literature [9,43]. The number of the average degrees of oligomerization (*P*_n_) were estimated from the mole fraction of unsaturated terminal chain ends, measured as the ratio between the total proton and vinylidene proton integrals in quantitative spectra.

## 3. Results and Discussion

**Figure 1:**
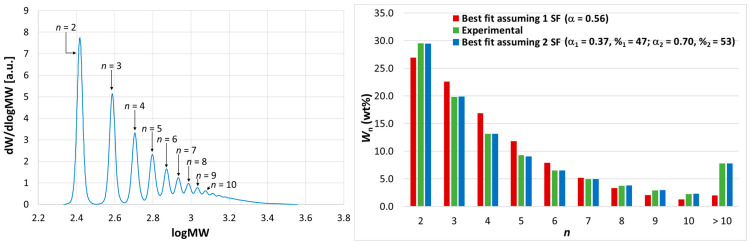
(**Left**) HR-HPLC trace of 1-octene oligomerization products obtained at 100 °C with **1**/MAO. Peak assignment is indicated explicitly (*n* = degree of oligomerization). (**Right**) Histogram comparing experimental MWD (blue bars) with best-fit MWDs built with one (red bars) or two (green bars) SF functions. See text, Table 1, and Table S1.

A first set of 1-octene oligomerization experiments was performed with **1**/MAO (Table 1, entries 1–16). As is typical with this activator (which also acts as a scavenger), catalyst productivity increased with an increase in the [Al]/[Zr] ratio (from 0.5 × 10^3^ to 2.0 × 10^3^). Experiments at varying reaction times highlighted the deactivating kinetics (see also Supporting Information, Figure S1). The HR-HPLC analysis of the products yielded a value of P_n_~3.5, independently of [Al]/[Zr], from which we concluded that the chain transfer by transalkylation with ‘free’ trimethylaluminum (TMA) was negligible. Consistently, the oligomer characterization by quantitative ^1^H NMR spectroscopy revealed the presence of large amounts of vinylidene groups, which are diagnostic of chain termination by β-H transfer (Figure S2) [43]. On the other hand, the traces (Figure 1-left) are incompatible with a single SF MWD, both for the absence of a maximum at the measured P_n_ value and the presence of a pronounced high-molar-mass tail instead. As a matter of fact, a good fit required a linear combination of two SF functions, with a roughly 1:1 mixing ratio and 2-fold different values of P_n_ (Figure 1-right and Table S1).

**Figure 2:**
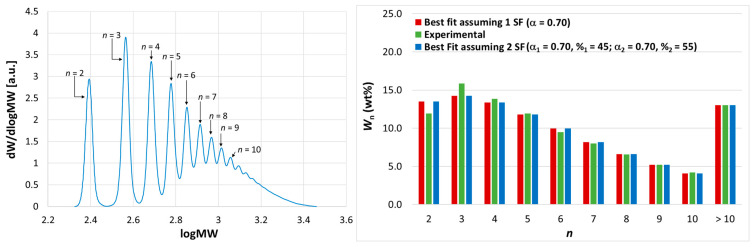
(**Left**) HR-HPLC trace of 1-octene oligomerization products obtained at 100 °C with **1**/TIBA/AB. Peak assignment is indicated explicitly (*n* = degree of oligomerization). (**Right**) Histogram comparing experimental MWDs (blue bars) with best-fit MWDs built with one (red bars) or two (green bars) SF functions. See text, Table 1, and Table S1.

Conversely, the HR-HPLC analysis of the oligomerization products made with catalyst systems **1**/TIBA/AB and **1**/TIBA/TTB (Table 1, entries 17–18 and 19–20, respectively) highlighted single SF distributions (as expected), with slightly higher *P*_n_ values compared with **1**/MAO and no tailing (Figure 2 and Table S1).

A series of oligomerization experiments were also carried out at *T* = 80 °C using MAO and TIBA/AB as cocatalysts and the results, in line with those obtained at 100 °C, are reported in the Supporting Information (Table S3 and Figure S3).

Although a broadening of the MWD possibly suggesting deviations from single-center behavior has been reported in the literature for polyethylene and polypropylene samples prepared with some MAO-activated metallocene catalysts [13,14], to the best of our knowledge, this is the first time that clear evidence of two discrete active species from one single metallocene precursor arising from MAO activation could be achieved, likely due to the oligomeric nature of the polyinsertion products and the high resolution of the HR-HPLC analysis. It is worth mentioning that no evidence of the formation of multiple active species deriving from the cyclometallation of the *n*BuCp fragment (e.g., Cp-terminated oligomers) was observed here [45,46,47].

The more likely explanation is that (at least) two active species, characterized by two slightly different α probabilities, are formed in situ despite the well-defined structure of the starting precatalyst [32]. Each of these active species would be therefore responsible for one of the SF distributions contributing to the overall product composition. Temperature variation might influence the relative abundance of the two species and/or the difference in their α probability, therefore explaining why the bimodal behavior is more evident at lower temperatures.

The above observations are in line with the hypothesis regarding multiple active species generating the bimodal distributions, which implies that, with borate activators, it is possible to selectively generate one active species which behaves similarly to one of the two formed with MAO (SI, Figure S3).

**Figure 3:**
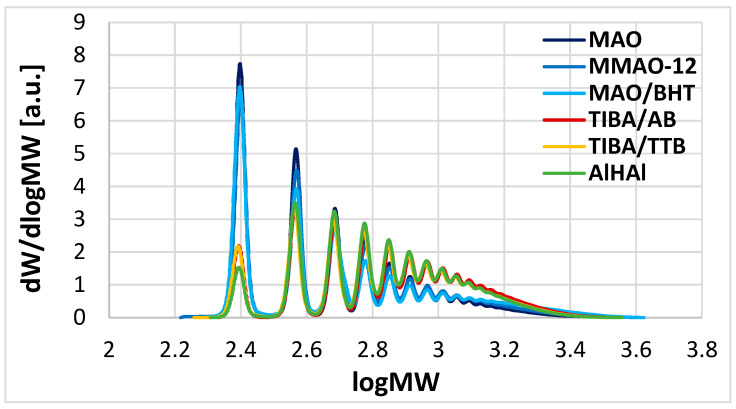
Overlay of HR-HPLC traces of several 1-octene oligomerization products of Table 1 obtained with **1** and various activators, namely MAO (entries 15–16), MMAO-12 (entries 21–22), MAO/BHT (entries 23–24), TIBA/AB (entries 17–18), TIBA/TTB (entries 19–20), and AlHAl (entries 25–26).

It is known that the complex and ill-defined structure of MAO contains Al centers of different Lewis acidity [48,49,50,51,52,53,54]; moreover, a certain amount of TMA is normally in equilibrium with the oligomeric part, and is often identified as the culprit for the in situ structural modification of the active species generated with MAO [26]. These may lead to the formation of multiple active species composed of the same cationic Zr species and various MAO-derived anions; different ion pairing effects between the cation and the various counterions may give rise to active species with slightly different propensities to react with the monomer [13,14,32,53]. Whether ion pairing effects or TMA binding to the active cation(s) is/are behind the diversification effects observed in catalyst speciation in the present case is an intriguing question that we addressed by extending the screening to other activators. Further 1-octene oligomerization experiments were carried out with MMAO-12 (Table 1, entries 21–22), MAO/BHT (Table 1, entries 23–24), and AlHAl (Table 1, entries 25–26). The overlays of HR-HPLC traces in Figure 3 and the fitting results shown in Table S1 indicate that MWDs consisting of two SF functions were invariably observed for products obtained with MAO-based activators, notwithstanding modifications aimed at reducing or suppressing the presence of ‘free’ TMA, whereas borate-based activators (including a novel one with a dinuclear hydride-bridged Al-alkyl cation, AlHAl) all gave products distributed according to single SF functions. We verified that the latter finding holds even when TMA is added on purpose to the catalyst system formulation (Table 1, entries 27–28).

Therefore, we trace the non-single-center nature of **1** following activation with MAO to ion pairing effects between the [(*n*BuCp)_2_Zr(Pol)]^+^ cation (Pol = Polymeryl) and the different negatively charged Al centers of MAO. We speculate that the consequences of small differences in the cation–anion binding strength (<1 kcal/mol, judging from the *P*_n_ values of the two SF distributions in Table S1) and therefore in the accessibility of the catalytic pocket were especially evident here, likely due to the high steric demand of the 2-hexyl-substituted olefin and Pol moiety.

**Figure 4:**
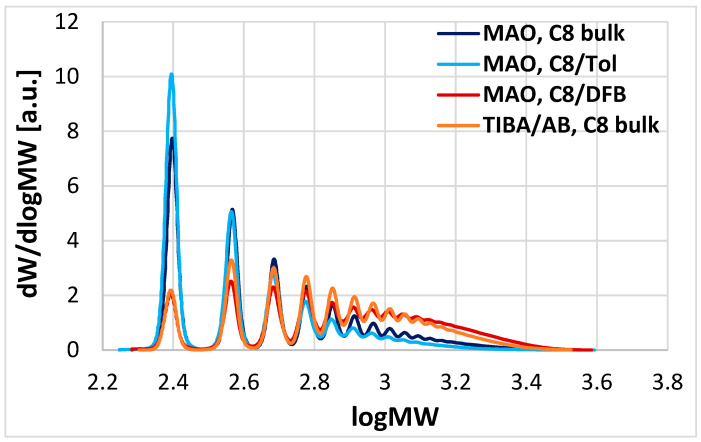
Overlay of HR-HPLC traces for several 1-octene oligomerization products in Table 1: Entries 15–16 (MAO activation, reaction in bulk 1-octene), 29–30 (MAO activation, reaction in 1-octene/DFB), 31–32 (MAO activation, reaction in 1-octene/toluene), 17–18 (TIBA/AB activation, reaction in 1-octene). See text for details.

To test the validity of our conclusion, we carried out two additional 1-octene oligomerization experiments diluting 1-octene (50/50 *v*/*v*) with toluene (dielectric constant *ε* = 2.4) and 1,2-difluorobenzene (*ε* = 13.8) both at 80 and 100 °C. From the results (Table 1, entries 29–32, Figure 4, and Figure S4) it can be seen on inspection that in polar medium, the MWD of the products conformed to a single SF function (and was indeed almost superimposable to that with **1**/TIBA/AB). On the other hand, diluting the monomer with toluene was inconsequential on product MWD, which remained bimodal. These observations represent strong supporting evidence for ion pairing being responsible for the bimodal distributions obtained with MAO in very low polarity media.

## 4. Conclusions

Ion pairing effects in α-olefin polymerizations mediated by Group 4 molecular (metallocene and ‘post-metallocene’) catalysts are quintessential. As a matter of fact, the serendipitous discovery of MAO and the key role of ‘poorly coordinating’ anions triggered the industrial application of those catalysts. On the other hand, cation–anion interactions in non-polar media are strong (7–13 kcal/mol, indicatively) [32] but rather nondirectional with the very weekly coordinating anions studied here. Moreover, the existence of discrete different ion pairs with an average persistence time longer than the polymer chain growth is rare; indeed, the MWD of the polyinsertion products typically conforms to the SF function.

In the present study, we found out that exceptions to the aforementioned scenario are possible, and that the speciation of an archetypal zirconocene precatalyst upon activation with MAO can originate (at least) two different catalytic species, each generating a SF MWD. In our opinion, the high steric demand of the monomer (1-octene) and, consequently, of the resulting polymer chain played an important role in this finding; however, the industrial relevance of the catalytic oligomerizations of bulky higher α-olefins is growing, and the impact of different activators on the product distribution for desired applications should be carefully considered.

## Figures and Tables

**Table 1:** Results of 1-octene oligomerization experiments at 100 °C in the presence of (pre)catalyst **1** and various activators (see text).

Entry	Activator	[Al]/[Zr] ×10^−2^	[B]/[Zr]	*T* (min)	Conv. (%)	Conv.,_av_ (%)	*R*_p_ ^(a)^	$\bar{Rp}$	*M*_n_ (Da)	$\bar{Mn}$	$\bar{Pn}$
1	MAO	5.0	-	30	2.7	2.7	3.1	3.2	342	347	3.1
2					2.8		3.3		351		
3	MAO	10	-	30	7.4	7.5	8.7	8.8	356	359	3.2
4					7.7		9.0		362		
5	MAO	20	-	5	4.6	4.2	32	29	452	459	4.1
6					3.7		26		465		
7	MAO	20	-	10	6.4	6.5	23	23	450	450	4.0
8					6.5		23		449		
9	MAO	20	-	20	8.0	10	14	16	417	422	3.8
10					11		19		426		
11	MAO	20	-	30	15	13	17	15	430	426	3.8
12					11		12		421		
13	MAO	20	-	45	20	20	16	15	423	421	3.8
14					19		15		418		
15	MAO	20	-	60	20	20	12	12	402	394	3.5
16					19		11		386		
17	TIBA/AB	2.0	2.0	60	16	16	9.3	8.9	528	537	4.8
18					15		8.5		546		
19	TIBA/TTB	2.0	2.0	60	22	23	14	13	568	572	5.1
20					23		13		575		
21	MMAO-12	20	-	60	23	23	13	14	411	419	3.7
22					24		14		427		
23	MAO/BHT ^(b)^	20	-	60	4.6	4.6	2.7	2.7	425	427	3.8
24					4.7		2.7		428		
25	AlHAl	0.10	5.0	60	4.9	4.6	2.8	2.6	592	595	5.3
26					4.3		2.5		598		
27	TIBA/TMA/AB ^(c)^	4.0	2.0	60	1.5	2.2	0.9	1.3	585	575	5.1
28					2.8		1.7		564		
29	MAO(DFB) ^(d)^	20	-	60	69	56	30	24	617	612	5.5
30					42		18		607		
31	MAO(Tol) ^(e)^	20	-	60	22	23	9.2	10	351	355	3.2
32					25		11		358		

^(a)^ In kg∙mmol_Zr_^−1^∙h^−1^. ^(b)^ [BHT]/[TMA] = 2. ^(c)^ [TIBA]/[TMA] = 1.0. ^(d)^ 1:1 *v*/*v* mixture of 1-octene and 1,2-difluorobenzene. ^(e)^ 1:1 *v*/*v* mixture of 1-octene and toluene.

## Data Availability

The original contributions presented in the study are included in the article and Supporting Information, further inquiries can be directed to the corresponding author.

## Supplementary Materials

The following supporting information can be downloaded at: https://www.mdpi.com/article/10.3390/polym17010046/s1, Figure S1: Monomer conversion vs. reaction time for the 1-octene oligomerization experiments; Figure S2: Overlay of the ^1^H NMR spectra of the samples produced at *T* = 100 °C with 1/MAO and 1/TIBA/AB; Table S1: Results of the simulation of experimental MWDs for selected 1-octene oligomerization products according to a one-SF or a two-SF model; Table S2: Experimental and fitted 1-octene oligomeric fractions in wt% for the products of Table S1; Table S3: 1-octene oligomerization experiments with various cocatalysts at 80 and 100 °C and *t* = 60 min; Figure S3: Top: Overlay of HPLC traces for the sample produced at 80 °C with MAO (blue) and TIBA/AB (orange); Bottom: Experimental vs. best-fit SF distributions for the sample produced at 80 °C with MAO (left) and TIBA/AB (right); Figure S4: Overlay of HPLC traces of the samples produced in bulk 1-octene with MAO and TIBA/AB and diluted 1-octene with MAO.
