# Supplementary material for: α-Olefin Oligomerization Mediated by Group 4 Metallocene Catalysts: An Extreme Manifestation of the Multisite Nature of Methylaluminoxane
Source: Polymers (Basel). 2024 Dec 28;17(1):46. doi: 10.3390/polym17010046 (PMC11723308; doi:10.3390/polym17010046)
Supplement: Supplementary file 1 [file polymers-17-00046-s001.zip › polymers-3368134-supplementary.pdf]

Supporting Information

α-Olefin oligomerization mediated by group 4 metallocene catalysts: an extreme manifestation of the multisite nature of methylaluminoxane

Francesco Zaccaria[1], Antonio Vittoria[1], Giuseppe Antinucci[1,], Roberta Cipullo[1,*], and Vincenzo Busico[1]*

[1] Department of Chemical Sciences, Federico II University of Naples, via Cinthia, 80126 Napoli, Italy

[*]Correspondence: giuseppe.antinucci@unina.it (G.A.); rcipullo@unina.it (R.C.)

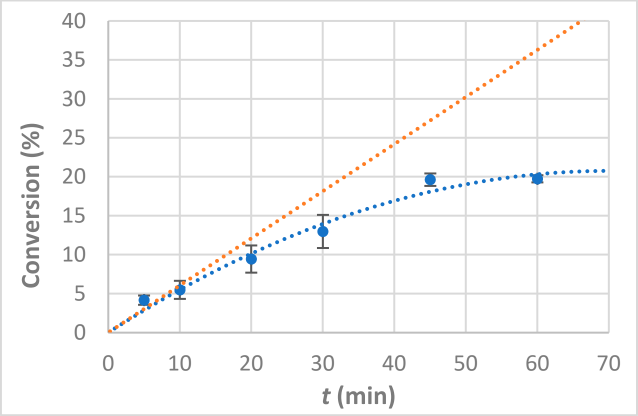

Figure S1. Monomer conversion *vs* reaction time for the 1-octene oligomerization experiments of Table 1, entries 5-16.

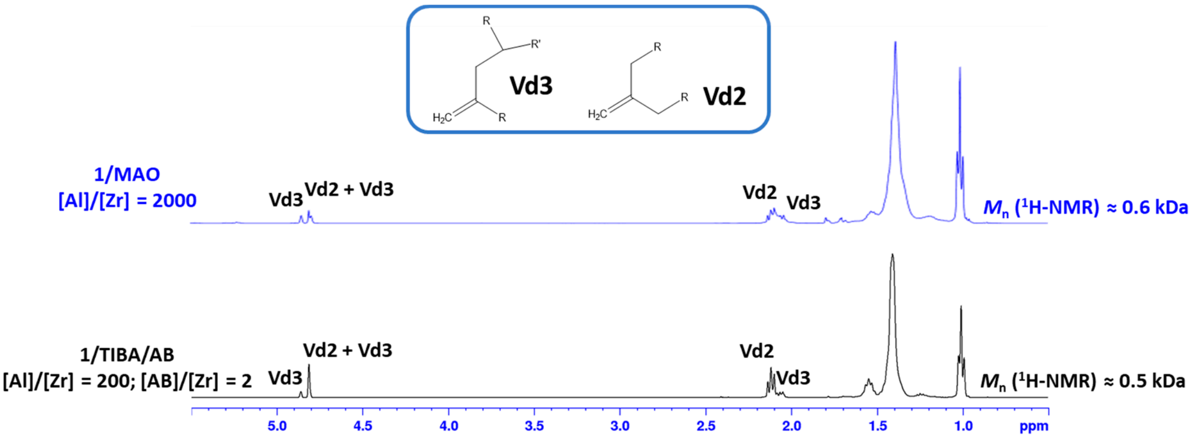

Figure S2. Overlay of the ^{1}H NMR spectra of the samples produced at T = 100°C with **1**/MAO and **1**/TIBA/AB (see Table 1). Unsaturated chain-end resonance attributions are based on the literature.[1]

Table S1. Results of the simulation of experimental MWD's for selected 1-octene oligomerization products in Table 1 according to a one-SF or a two-SF model (see text; best-fitting results highlighted in bold).

Entry	Activator	One-SF model			Two-SF model						
		α[(a)]	P_n	χ_R^2[(b)]	α_1[(a)]	$P_{n,1}$	$\%_1$	α_2[(a)]	$P_{n,2}$	$\%_2$	χ_R^2[(b)]
8	MAO	0.56	2.3	8	**0.37**	**1.6**	**47**	**0.70**	**3.5**	**53**	**0.02**
11	MMAO-12	0.60	2.5	11	**0.37**	**1.6**	**44**	**0.74**	**3.8**	**56**	**0.1**
12	MAO/BHT	0.58	2.4	25	**0.37**	**1.6**	**52**	**0.78**	**4.6**	**48**	**2.2**
9	TIBA/AB	**0.70**	**3.4**	**0.6**	0.70	3.4	50	0.70	3.4	50	1.0
10	TIBA/TTB	**0.73**	**3.7**	**0.8**	0.73	3.7	50	0.73	3.7	50	1.2
13	Al-H-Al	**0.74**	**3.8**	**3.2**	0.74	3.8	50	0.74	3.8	50	4.9

[(a)] Stochastic probability of chain propagation. [(b)]Value of the reduced-chi^2 function of the model.

Table S2. Experimental and fitted 1-octene oligomeric fractions in wt% for the products of Table S1.

Entry	Activator	*n*	Experimental (wt%)	1 SF model	2 SF model
			wt%	*wt%*	*wt%*
8	MAO	2	29.5	26.9	29.5
		3	19.9	22.6	19.9
		4	13.1	16.9	13.2
		5	9.3	11.8	9.1
		6	6.5	7.9	6.6
		7	5.0	5.2	4.9
		8	3.7	3.3	3.8
		9	2.9	2.1	3.0
		10	2.2	1.3	2.3
		> 10	7.8	2.0	7.8
9	TIBA/AB	2	11.9	13.5	13.5
		3	15.8	14.3	14.3
		4	13.9	13.4	13.4
		5	11.9	11.8	11.8
		6	9.5	10.0	10.0
		7	8.0	8.2	8.2
		8	6.5	6.6	6.6
		9	5.2	5.2	5.2
		10	4.2	4.1	4.1
		> 10	13.0	13.0	13.0
10	TIBA/TTB	2	8.9	11.4	11.4
		3	13.8	12.5	12.5
		4	12.9	12.2	12.2
		5	11.6	11.1	11.1
		6	9.6	9.8	9.8
		7	8.3	8.3	8.3
		8	7.1	7.0	7.0
		9	5.9	5.7	5.7
		10	4.7	4.6	4.6
		> 10	17.2	17.3	17.3

Table S3. 1-octene oligomerization experiments with various cocatalysts at 80 and 100°C and t = 60 min.

Exp.	T (°C)	Cocatalyst	[B]/[Zr]	[Al]/[Zr]	Conv. (%)	R_p ($kg \cdot mmol_{Zr}^{-1} \cdot h^{-1}$)	M_n (Da)	M_w (Da)	PDI
1	100	MAO	-	2000	19.7	11.6	394	572	1.5
2	80	MAO	-	2000	15.2	8.9	583	940	1.6
3	100	AB/TIBA	2	200	15.2	8.9	537	738	1.4
4	80	AB/TIBA	2	200	11.5	6.7	955	1473	1.5

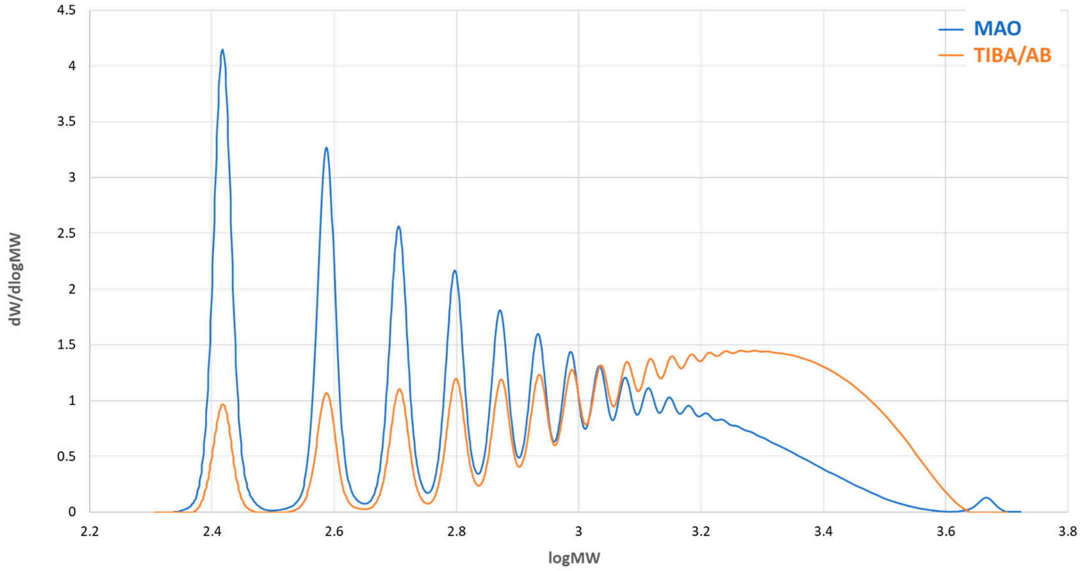

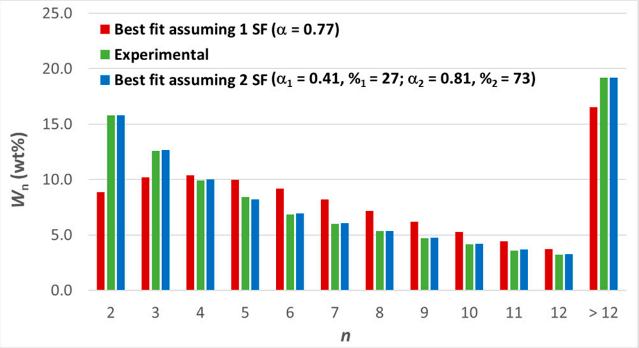

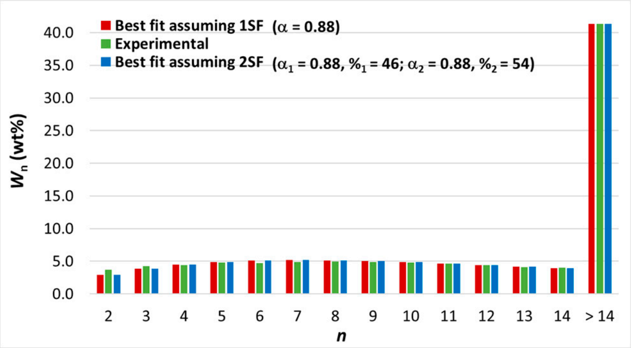

Figure S3. Top: Overlay of HPLC traces for the sample produced at 80°C with MAO (blue) and TIBA/AB (orange); Bottom: Experimental *vs* best-fit SF distributions for the sample produced at 80 °C with MAO (left) and TIBA/AB (right).

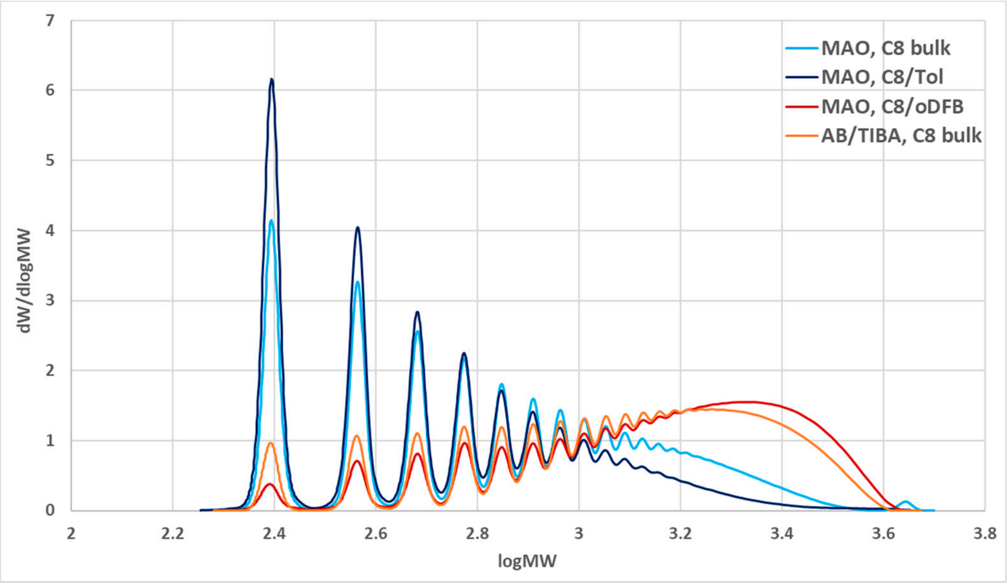

Figure S4. Overlay of HPLC traces of the samples produced in bulk 1-octene with MAO and TIBA/AB and diluted 1-octene with MAO.

References

[1] V. Busico, R. Cipullo, N. Friederichs, H. Linssen, A. Segre, V. Van Axel Castelli, G. van der Velden, H NMR Analysis of Chain Unsaturations in Ethene/1-Octene Copolymers Prepared with Metallocene Catalysts at High Temperature. *Macromolecules* **2005**, *38*, 6988.
